# Homogeneous, Synthetic, Non-Saccharide Glycosaminoglycan Mimetics as Potent Inhibitors of Human Cathepsin G

**DOI:** 10.3390/biom13050760

**Published:** 2023-04-27

**Authors:** Daniel K. Afosah, Rawan M. Fayyad, Valerie R. Puliafico, Spencer Merrell, Eltice K. Langmia, Sophie R. Diagne, Rami A. Al-Horani, Umesh R. Desai

**Affiliations:** 1 Department of Medicinal Chemistry, School of Pharmacy, Virginia Commonwealth University, Richmond, VA 23298, USA; fayyadr@vcu.edu (R.M.F.);; 2 Institute for Structural Biology, Drug Discovery and Development, Virginia Commonwealth University, Richmond, VA 23219, USA; 3 Department of Chemistry and Biochemistry, Washington and Lee University, Lexington, VA 24450, USA; 4 Department of Chemistry, Virginia Commonwealth University, Richmond, VA 23298, USA; 5 Division of Basic Pharmaceutical Sciences, College of Pharmacy, Xavier University of Louisiana, New Orleans, LA 70125, USA

**Keywords:** allosterism, cathepsin G, glycosaminoglycans, mimetics, inflammation

## Abstract

Cathepsin G (CatG) is a pro-inflammatory neutrophil serine protease that is important for host defense, and has been implicated in several inflammatory disorders. Hence, inhibition of CatG holds much therapeutic potential; however, only a few inhibitors have been identified to date, and none have reached clinical trials. Of these, heparin is a well-known inhibitor of CatG, but its heterogeneity and bleeding risk reduce its clinical potential. We reasoned that synthetic small mimetics of heparin, labeled as non-saccharide glycosaminoglycan mimetics (NSGMs), would exhibit potent CatG inhibition while being devoid of bleeding risks associated with heparin. Hence, we screened a focused library of 30 NSGMs for CatG inhibition using a chromogenic substrate hydrolysis assay and identified nano- to micro-molar inhibitors with varying levels of efficacy. Of these, a structurally-defined, octasulfated di-quercetin NSGM **25** inhibited CatG with a potency of ~50 nM. NSGM **25** binds to CatG in an allosteric site through an approximately equal contribution of ionic and nonionic forces. Octasulfated **25** exhibits no impact on human plasma clotting, suggesting minimal bleeding risk. Considering that octasulfated **25** also potently inhibits two other pro-inflammatory proteases, human neutrophil elastase and human plasmin, the current results imply the possibility of a multi-pronged anti-inflammatory approach in which these proteases are likely to simultaneously likely combat important conditions, e.g., rheumatoid arthritis, emphysema, or cystic fibrosis, with minimal bleeding risk.

## 1. Introduction

**Figure 1:**
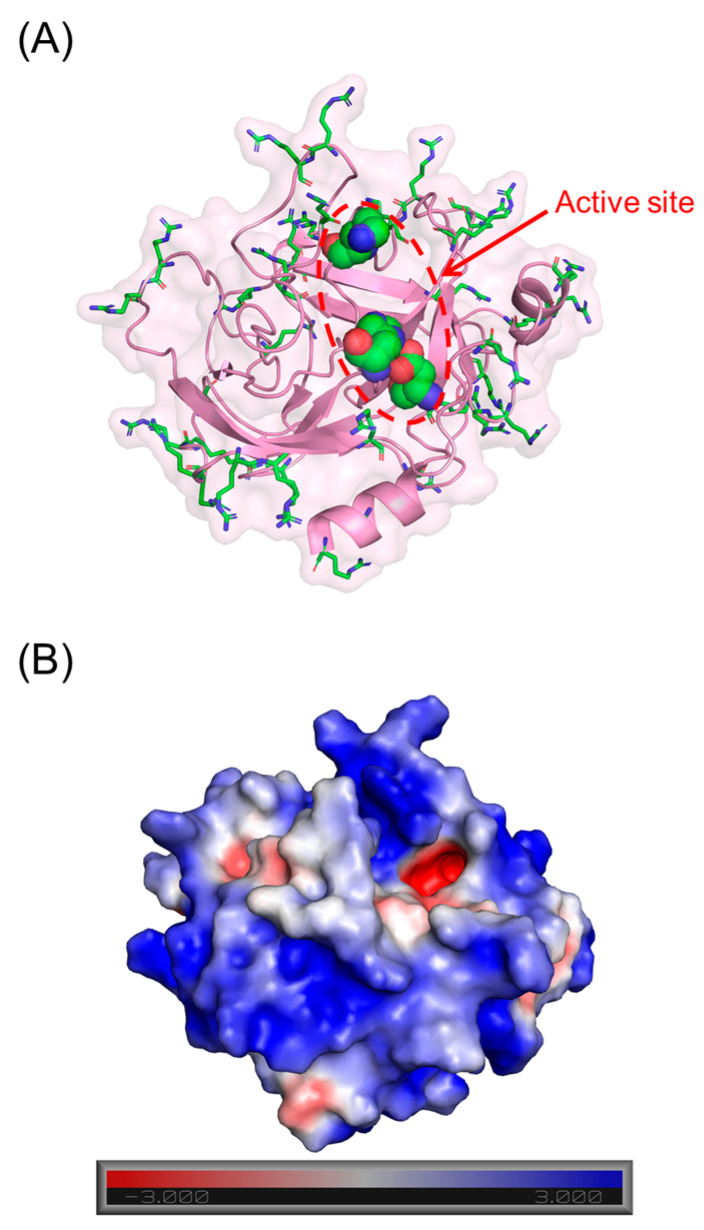
Structure of human CatG (PDB ID = 1 KYN). (**A**) shows that basic (sticks) and catalytic triad residues (spheres) of CatG include His57, Asp102, and Ser195 residues (chymotrypsin numbering). (**B**) shows the nature of electrostatic surface of the protease. Red and blue represent electronegative and electropositive surfaces, respectively.

Human cathepsin G (CatG) is a member of the neutrophil serine proteases (NSPs), a group of proteins released from the azurophilic granules of neutrophils. These proteases, which also include human neutrophil elastase (HNE), proteinase 3 (PR3), and neutrophil serine protease 4 (NSP4), are most associated with the host defense mechanism against pathogens [1]. Mature CatG is made up of 235 residues [2], and similar to the other NSPs, has an overall positive charge due to an abundance of basic residues (Figure 1) [3]. CatG has broad substrate specificity which affords additional roles, including the degradation of extracellular matrix; activation of matrix metalloproteases; processing and release of cytokines, chemokines, and growth factors; activation of caspases; generation of angiotensin II; and activation of platelet receptors [4,5,6,7,8,9,10,11,12]. Owing to this, nature has devised several physiological inhibitors, including α_1_-antichymotrypsin, α_1_-proteinase inhibitor, α_2_-macroglobulin, serpin B1, proteinase inhibitor, and secretory leukocyte protease inhibitor, that regulate its activity [13]. Recently, the role of NSPs in the inflammatory process has garnered a lot of attention because it is becoming clear that runaway inflammation arises from dysregulation of the natural protease–antiprotease balance [14,15,16,17].

CatG plays important roles in multiple inflammatory diseases including, rheumatoid arthritis [18], psoriasis [19,20], chronic obstructive pulmonary disease (COPD) [21,22], emphysema [23], and cystic fibrosis (CF) [24]. Elevated CatG expression is one of the causative disruptors of the natural protease–antiprotease balance, and is typically observed in these conditions. More importantly, high enzyme activity sets in motion the degradation of the connective tissue and ECM proteins, while also promoting apoptosis and activation of other pro-inflammatory mediators [13]. In fact, CatG has been identified as a marker of airway inflammation and a predictor of disease progression in COPD [25]. Thus, small molecule or biologic inhibitors of CatG are expected to be very useful. Unfortunately, no FDA-approved inhibitor of CatG is available to date. In fact, only a few CatG inhibitors have been reported so far, such as oligopeptides, small molecules, and aptamers [13,26,27,28]. Of these, none have reached clinical trials [13].

Glycosaminoglycans (GAGs), especially heparin, have been reported to inhibit CatG activity [29,30]. Although recently Burster et al. state in their review that the ‘characteristics of heparin to modulate the activity of CatG are controversial and might depend on the heparin concentration’ [31], the promise of heparin has been that it has been known to modulate many pro-inflammatory proteases for a long time [32,33,34,35,36]. However, the tendency to induce bleeding and considerable heterogeneity limits its use in a majority of diseases. In this context, small sulfated molecules that mimic heparin function may represent a major avenue for discovering novel therapeutics. Whereas mimicking is typically regarded as structural in nature, we have shown over the past decade that small sulfated molecules mimic function because of the presence of multiple sulfate groups on the small scaffold.

One such small molecule, labeled as sulfated pentagalloyl glucoside (SPGG), a member of the library of non-saccharide GAG mimetics (NSGMs), was recently reported as an allosteric inhibitor of CatG [37]. Unfortunately, SPGG is also a heterogeneous mixture composed of variably-sulfated species [38]. Additionally, it inhibits human factor XIa and induces powerful blood anticoagulation [39], which could induce some bleeding risk. We reasoned that it should be possible to discover structurally-defined and homogeneous molecules that inhibit CatG with high potency and present no or minimal anticoagulation potential by screening the library of NSGMs. In this work, we screened a focused library of 30 NSGMs and identified an octasulfated **25** as a potent inhibitor of CatG (IC_50_ 53 nM). As expected, NSGM **25** was found to be an allosteric inhibitor of CatG. Interestingly, salt-dependence studies indicated that NSGM **25** utilized almost equal ionic and nonionic forces in binding to CatG, which alludes to the special role of the aromatic scaffold of these GAG mimetics. NSGM **25** did not extend human plasma clotting time in the activated partial thromboplastin time and prothrombin time assays, suggesting the strong possibility of no or minimal bleeding complications. Overall, this work presents at least one novel synthetic, homogeneous small molecule as a nanomolar allosteric inhibitor of CatG, devoid of anticoagulant properties.

## 2. Materials and Methods

### 2.1. Materials

Human CatG and chromogenic substrate for CatG (S-7388, *N*-succinyl-Ala-Ala-Pro-Phe *p*-nitroanilide) were purchased from Elastin Products Company (Owensville, MO, USA). Human plasma was obtained from George King Biomedical Inc. (Overland Park, KS, USA). Stock solutions of CatG were prepared in 20 mM tris-HCl buffer, pH 7.4, containing 0.02% Tween 80, 0.1% PEG 8000, 2.5 mM CaCl_2_, and 100 mM NaCl. Experiments were repeated at least two times.

### 2.2. Chemistry

All the molecules in this study were characterized by NMR, MS, and UPLC, and reported to have purity of >95%. The synthetic schemes and spectral data of all molecules studied in this work have been previously reported [40,41,42].

### 2.3. Inhibitor Screen

Using a chromogenic substrate hydrolysis assay, compounds were screened at a concentration of 50 µM, employing a 96-well plate format. To each well of a 96-well microplate containing 88 µL of 20 mM tris buffer, which contained 100 mM NaCl, 2.5 mM CaCl_2_, 0.1% PEG 8000, and 0.05% Tween 80, 4 µL of CatG (final concentration of 60 nM) and 5 µL of H_2_O or NSGM (final concentration of 50 µM) were added. After incubating for a period of 5 min, 3 µL of CatG substrate (S-7388, final concentration of 750 µM) was added, and the residual CatG activity was obtained from the initial rate of increase of absorbance at 405 nm. The relative residual activity of CatG for each of the NSGMs, at the various concentrations, was calculated from the ratio of CatG activity in the presence and absence of NSGMs. Each compound was tested twice and compounds that showed greater than 50% inhibition of CatG were selected for IC_50_ determination.

### 2.4. IC_50_ Determination

A chromogenic substrate hydrolysis assay was used to determine the direct inhibition of CatG by the NSGMs, as previously reported [37]. To each well of a 96-well microplate containing 88 µL of 20 mM tris buffer, containing 100 mM NaCl, 2.5 mM CaCl_2_, 0.1% PEG 8000, and 0.05% Tween 80, 4 µL of CatG (final concentration of 60 nM) and 5 µL of H_2_O or NSGM (final concentration of 0–100 µM) were added. After incubating for a period of 5 min, 3 µL of CatG substrate (S-7388, final concentration of 750 µM) was added, and the residual CatG activity was obtained from the initial rate of increase of absorbance at 405 nm. The relative residual activity of CatG for each of the NSGMs, at the various concentrations, was calculated from the ratio of CatG activity in the presence and absence of NSGMs. The dose dependence curve was plotted using a logistic equation (see below) in Sigmaplot version 12 to obtain the IC_50_ (potency), Hill slope (HS), and efficacy (ΔY). Here, Y is the ratio of residual CatG activity in the presence of NSGMs to that in their absence, Y_O_ and Y_M_ are the minimum and maximum values of fractional residual CatG activity, respectively, IC_50_ is the concentration of the NSGM that inhibits CatG activity by 50%, and HS is the Hill slope. ΔY = Y_M_ − Y_O_.

$$Y=Y_{O}+\frac{Y_{M}-Y_{O}}{1+10^{\left(\log{\left[I\right]}_{0}-{\mathrm{logIC}}_{50}\right)\left(\mathrm{HS}\right)}}$$

### 2.5. Michaelis–Menten Kinetics

The initial rate of the hydrolysis of CatG substrate by NSGM **25** was monitored using the linear increase in absorbance corresponding to less than 10% consumption of substrate at 37 °C in pH 7.4 20 mM tris buffer containing 100 mM NaCl, 2.5 mM CaCl_2_, 0.1% PEG 8000, and 0.05% Tween 80, as for IC_50_ determinations. The initial rate was measured at various substrate concentrations (0–2500 μM) at fixed enzyme concentration (100 nM) and fixed inhibitor concentrations (0–200 nM). The data were analyzed using the standard Michaelis–Menten equation in Sigmaplot version 12 to determine the K_M_ and V_MAX_.

### 2.6. Salt Dependence of NSGM ***25*** Inhibition of CatG

The direct inhibition of CatG cleavage of a chromogenic substrate was determined at 37 °C, as described above, in pH 7.4 tris buffer containing 2.5 mM CaCl_2_, 0.1% PEG 8000, 0.05% Tween 80, and 50–200 mM NaCl. Each K_I_ value was calculated from the corresponding IC_50_ using the Cheng–Prusoff equation [43]. A double-log plot of the K_I_ against Na^+^ concentration was prepared and analyzed using the equation ${\log\;K}_{I}={\log\;K}_{I,\mathrm{NONIONIC}}+Z\psi \log\left[{\mathrm{Na}}^{+}\right]$. Here, the slope corresponds to the number of ion-pair interactions (Z) and the counterions released per negative charge upon ion binding (ψ = 0.8), while the intercept corresponds to the nonionic affinity (K_I,NONIONIC_). The contributions of ionic and nonionic binding energies to the interactions were obtained from the slope and intercept of the plot in Excel.

### 2.7. Impact of NSGM ***25*** on Clotting Assays

The impacts of NSGM **25** on activated partial thromboplastin time (aPTT) and the prothrombin time (PT) of human plasma were measured employing a standard one-stage recalcification assay at 37 °C, as previously reported [44]. CaCl_2_ and thromboplastin-D were used to initiate clotting in aPTT and PT assays, respectively, in the absence and presence of NSGM **25**, and the time to clot was recorded accordingly.

## 3. Results

### 3.1. Screening for Cathepsin G Inhibition

**Figure 2:**
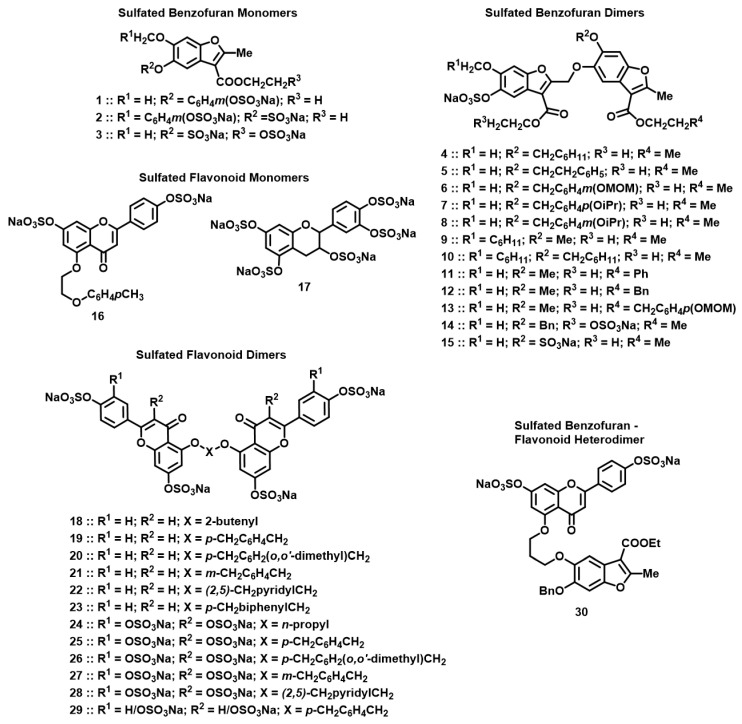
Chemical structures of the focused library of non-saccharide glycosaminoglycan mimetics (NSGMs). Structures studied included sulfated benzofuran monomers **1**–**3** (see Table 1 for R^1^, R^2^, R^3^); sulfated benzofuran dimers **4**–**15** (see Table 1 for R^1^, R^2^, R^3^ and R^4^); sulfated flavonoid monomers **16** and **17**; sulfated flavonoid dimers **18**–**29** (see Table 2 for X, R^1^, and R^2^); and sulfated benzofuran–apigenin heterodimer **30**.

The library of NSGMs consisted of 30 synthetic, sulfated compounds, based on either a benzofuran or flavonoid scaffold with varying level and pattern of sulfate groups (Figure 2, Table 1 and Table 2). Both benzofuran- and flavonoid-based NSGMs have been studied earlier for anticoagulant [40,45,46], antiviral [41], and antifibrinolytic activities [47]. More importantly, we reasoned that the configurational and conformational diversity afforded by these NSGMs is sufficiently broad to afford a high probability of initial hits that could later be transformed into CatG selective agents. The NSGMs were either monomers or homo-/hetero-dimers that presented a linear molecular length of ~13 to 30 Å, which is the length of a typical heparin-binding site on proteins.

**Figure 3:**
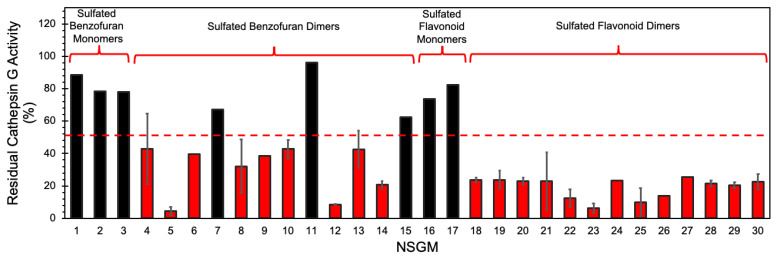
Screening of focused chemical library of NSGMs against CatG. CatG inhibition was measured using a chromogenic substrate hydrolysis assay in a 20 mM tris buffer, pH 7.4, containing 100 mM NaCl, 2.5 mM CaCl_2_, 0.1% PEG, and 0.05% Tween 80 at 37 °C. Compounds were screened at 50 µM and measurements were performed at least in duplicate. Error bars represent ± 1 S.E. Red bars show compounds selected for *IC*_50_ determination. Black bars show compounds with <50% inhibition and identified as weak/modest inhibitors of CatG.

We utilized a screening strategy involving the use of a chromogenic substrate (S-7388), which had been used earlier in multiple studies [37]. We first screened the NSGMs for CatG inhibition at 50 µM in a pH 7.4 tris buffer containing 100 mM NaCl, 2.5 mM CaCl_2_, 0.1% PEG, and 0.05% Tween 80 to quickly assess the diversity of CatG targeting [37]. Indeed, Figure 3 shows a massive structural dependence of activity, with 2–95% inhibition of CatG. Of these, monomeric NSGMs were found to be poor CatG inhibitors, with none displaying more than 50% inhibition at 50 µM. In contrast, nearly all dimeric NSGMs exhibited significant CatG inhibition (>50%). This phenomenon is similar to earlier results for many NSGM–protein systems [40,48], and correlates with the observation that longer GAGs elicit better biological responses.

### 3.2. Structure–Activity Relationship (SAR)

**Figure 4:**
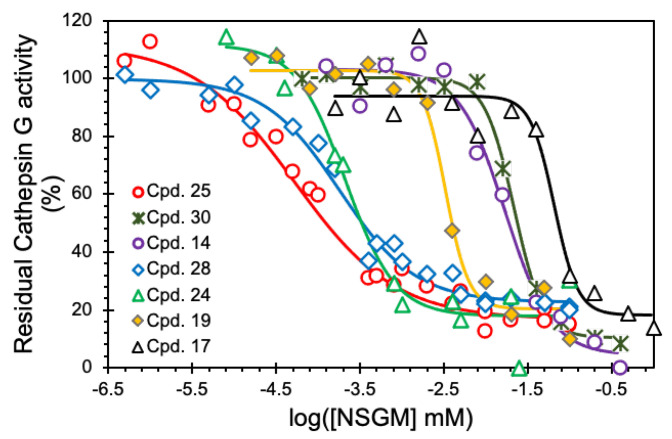
Representative IC_50_ profiles for CatG inhibition by NSGMs. Direct inhibition of human CatG was measured using the chromogenic substrate hydrolysis assay at pH 7.4 and 37 °C. Solid lines represent analysis using the logistic dose–response relationship (Equation (1)) to obtain the IC_50_, *ΔY%*, and HS. Errors represent ± 1 S.E.

To identify promising NSGMs, we measured the IC_50_ of 22 NSGMs that inhibited CatG by at least 50%. Figure 4 shows sigmoidal dose–response relationships for a select group of NSGMs, suggesting nearly a 1000-fold range of potencies. While sulfated benzofuran dimers **4**–**15** displayed IC_50_s in the range of 5 to >50 µM (Table 1), sulfated flavonoid dimers presented IC_50_ in the range of 0.05–10 µM (Table 2). At first glance, this could represent a preference for the flavonoid scaffold, but such a generalization would be inaccurate because there is a vast difference in the level of sulfation between the two scaffolds. While the benzofuran dimers had only one or two sulfate moieties, the flavonoid dimers had a minimum of four sulfates. More interestingly, the level of sulfation, although necessary and important for binding to electropositive CatG (Figure 1), appears to contribute additional factors, which are described below.

**Benzofuran-based NSGMs—**Although sulfated benzofuran NSGMs displayed modest IC_50_s (5.5 to >50 µM, Table 1), interesting insights can be derived in terms of CatG recognition. CatG is a strongly basic protease with a predicted pI of 12 [49] and a predominantly electropositive surface area (Figure 1). A priori, this implies a high probability of binding to polyanionic species such as long chain GAGs. Nevertheless, mono- and di-sulfated benzofurans, consisting of hydrophobic and aromatic groups, were found to be modest inhibitors of CatG. In fact, the hydrophobic substituents, rather than the number of sulfate moieties, appeared to be more important for CatG inhibition in this series. This implies that the monosulfated benzofuran scaffold may serve as a useful fragment for conjugation with a promising hit from another screen.

This is not the first time that hydrophobic scaffolds and/or substituents of highly-sulfated NSGMs have been found to induce inhibition of serine proteases. In fact, an optimal combination of hydrophobic and anionic forces was proposed to be the basis for both affinity and selectivity of sulfated NSGMs, as described in a recent perspective [42,50,51]. More specifically, whereas sulfated benzofuran dimer **5**, having a phenethyl group at the R^2^ position, displayed an IC_50_ of 5.5 µM, dimer **15**, with a sulfate group in the same position, was more than 10-fold less potent (Table 1). However, the presence of a substituted aromatic ring in the same position, e.g., dimers **6**–**8**, resulted in at least a five-fold loss in potency. Introducing an alicyclic ring, as in dimer **4**, at the same position (R^2^) led to a nine-fold loss in potency. These results suggest that the phenethyl group at the R^2^ position occupies a well-defined pocket that contributes to the selectivity of binding.

The presence of a bulky hydrophobic group at the R^1^ position of sulfated benzofuran dimers appears to marginally favor CatG inhibitory potency as evidenced by NSGM **10** (IC_50_ 29 µM), which was almost two-fold more potent than NSGM **4** (Table 1). Likewise, the benzyl group at the R^4^ position (dimer **12**) is slightly favored over phenyl (dimer **11**) or substituted phenyl group (dimer **13**). Of note, both of these observations convey the importance of the nature of hydrophobic groups in improving potency. Interestingly, enhancing sulfation level did not offer better inhibition potential over monosulfated benzofuran dimer **5**, as evidenced by disulfated NSGMs **14** and **15**. In fact, **14** having an additional sulfate at the R^3^ position was nearly three-fold less potent than dimer **5**, while NSGM **15** was essentially inactive (IC_50_ > 50 µM, Table 1).

Overall, these results for sulfated benzofuran dimers underscore the importance of the aromatic/hydrophobic groups at the R^2^ position, while also emphasizing the importance of the positions, rather than the number, of sulfate groups for CatG inhibition. The SAR observed for this series of inhibitor hits suggests that appropriate modification or conjugation at the R^1^ and R^3^ positions may significantly increase inhibition potency. From a drug design/discovery perspective, such modifications are much easier to introduce than altering the number and position of sulfate groups.

**Flavonoid-based NSGMs—**This sub-library of dimers is made up of di-quercetin, quercetin–apigenin, and di-apigenin dimers with 8, 6, and 4 sulfate groups, respectively. Alternatively, this class of NSGMs was completely different from the sulfated benzofuran dimers in terms of both the scaffold as well as the level of sulfation. Table 2 lists the inhibition potencies of NSGMs **18**–**29**, which present a fairly wide range, from 0.05 to 10.3 μM. Interestingly, as a group, tetra-sulfated NSGMs, e.g., **18**–**23**, were ~50-fold less potent than octasulfated NSGMs, e.g., **24**–**28**. Alternatively, this group of NSGMs presents the conclusion that higher sulfation is better for CatG inhibition, an observation directly opposed to the results presented above for NSGMs **4**–**15** (Table 1). More specifically, comparing NSGMs with identical linkers, e.g., **25** (IC_50_ 0.05 µM) vs. **19** (IC_50_ 3.3 µM), shows a 66-fold difference between octasulfated di-quercetin NSGM and its tetra-sulfated di-apigenin counterpart.

For the di-apigenin-based NSGMs, the most potent molecule, **22** (IC_50_ 1.3 µM), had a 2,6-bis(methylene)pyridine linker (Table 2). Substitution of the linker with a 1,3-bis(methylene)benzene in NSGM **21** resulted in an eight-fold decrease in potency, possibly indicating a role for the heteroatom in binding. A change from meta- to para-substitution in the linker, i.e., **21** vs. **19**, resulted in a two-fold loss. This implies that meta to para change probably impacts the relative spatial arrangement of each monomer as well as their sulfates.

For the di-quercetin-based NSGMs, the most potent NSGM, **25** (IC_50_ 0.05 µM), had a 1,4-bis(methylene)benzene linker. Introducing methyl groups on a linker aryl ring (NSGM **26**) did not affect CatG inhibitory potency; however, a three-fold decrease in potency was observed when the linker was changed to 1,3-bis(methylene)benzene (NSGM **27**, IC_50_ 0.15 µM). This is similar to what was observed for the di-apigenin-based NSGMs (above). Contrary to the observation with di-apigenin dimer **22**, the presence of a heteroatom in the linker in di-quercetin dimer **28** had no impact on potency when compared with respective parent NSGMs (e.g., **21** and **27**). Introducing a more flexible linker induced a 4-fold loss of potency (i.e., **25** vs. **24**), and suggests a possibly-limited ability to maneuver around the linker structure.

Finally, the most convincing evidence of the importance of the linker is seen with NSGM **29**, a hexa-sulfated quercetin–apigenin heterodimer carrying a 1,4-bis(methylene)benzene linker. NSGM **29** displayed an IC_50_ of 0.14 µM, which is several-fold lower than the di-apigenin-based NSGMs (1.3–10.3 µM), but only 2.8-fold higher than the most potent di-quercetin NSGM **25** (0.05 µM). In fact, heterodimer **29** is equipotent with most di-quercetin NSGMs, despite having two fewer sulfate groups. This implies that there is enough structural space available around the quercetin–apigenin heterodimeric scaffold for the discovery of more potent leads, if needed.

### 3.3. Mechanism of CatG Inhibition by NSGM ***25***

GAGs typically engage proteases, especially coagulation factors, in their allosteric sites as discussed in recent articles [51,52]. Because NSGMs are known to functionally mimic GAGs, allosterism is also expected and observed, especially with regard to coagulation proteases [52]. However, structurally, NSGMs present a combination of a hydrophobic and an anionic scaffold, while GAGs are strongly anionic, which may induce different binding sites and/or geometries for some proteins. In fact, one example of this phenomenon has already been documented. A monosulfated benzofuran-based NSGM inhibited thrombin by binding in a locale different from that of a heparan sulfate oligosaccharide [53]. Thus, it is important to assess the mechanism of inhibition induced by NSGMs, especially the most promising molecules, for every target protease. The most promising molecule in this work was found to be NSGM **25** (IC_50_ = 50 nM), which is the reason mechanistic studies were performed for this representative inhibitor.

**Figure 5:**
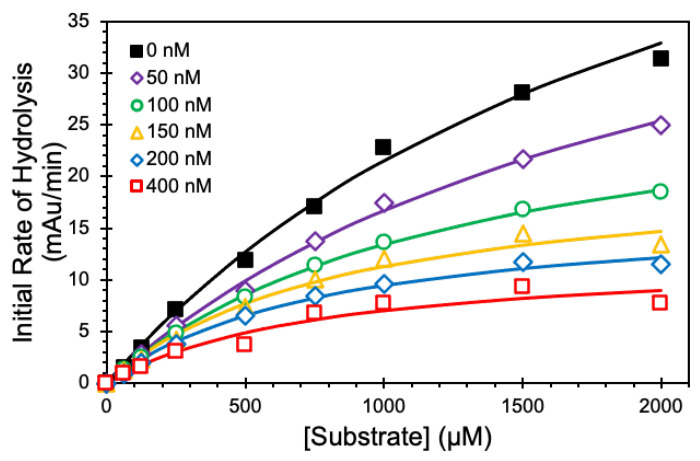
Kinetics of inhibition of 60 nM CatG by NSGM **25**. Michaelis–Menten kinetics of CatG hydrolysis of chromogenic substrate (S-7388) in the presence of NSGM **25**. Experiments were performed at 37 °C in 20 mM tris buffer, pH 7.4. Solid lines represent nonlinear regression analysis of the data using the standard Michaelis-Menten to calculate K_M_ and V_MAX_.

We employed Michaelis–Menten kinetic studies to define the mechanism of CatG inhibition by NSGM **25** at pH 7.4 and 37 °C (Figure 5). In the absence of **25**, the K_M_ for the substrate was 2.26 ± 0.28 mM. As the concentration of **25** increased from 50 to 400 nM, the K_M_ decreased consistently and reached a value of 0.8 ± 0.3 mM (Table 3). Similarly, there was a corresponding decrease in the V_MAX_ from 70 ± 6 to 12 ± 2 mAu/min. The simultaneous reductions in both K_M_ and V_MAX_ suggest an uncompetitive inhibition mechanism, a type of allosteric inhibition where the inhibitor preferentially binds only to the substrate-bound protease to bring about inhibition.

### 3.4. Salt-Dependence of CatG Inhibition in the Presence of NSGM ***25***

A fundamental point being advanced in NSGM-based mimicry is the presumed increase in hydrophobic forces contributing to binding affinity. As is well-recognized, heparin and other GAGs utilize primarily electrostatic forces in binding to proteins [54]. Only when the contribution of nonionic forces, e.g., van der Waals, and/or directional ionic forces, e.g., hydrogen bonding (H-bonding), is high enough do GAGs exhibit a high level of selectivity. This is exemplified by the classic case of antithrombin binding to heparin, which exhibits nearly 60% nonionic binding energy [55,56]. The resolution of overall binding energy into ionic and nonionic contributions is typically achieved by performing affinity measurements as a function of the ionic strength of the buffer. According to the protein–polyelectrolyte theory [57], the two contributions can be resolved from a double-log plot of binding affinity against the Na^+^ concentration, as defined by the equation log K_I_ = log K_I,NONIONIC_ + Zψ × log [Na^+^], where Z represents the number of salt interactions, and ψ is the proportion of Na^+^ released per anion upon ligand binding and is equal to 0.8 for heparin [55].

**Figure 6:**
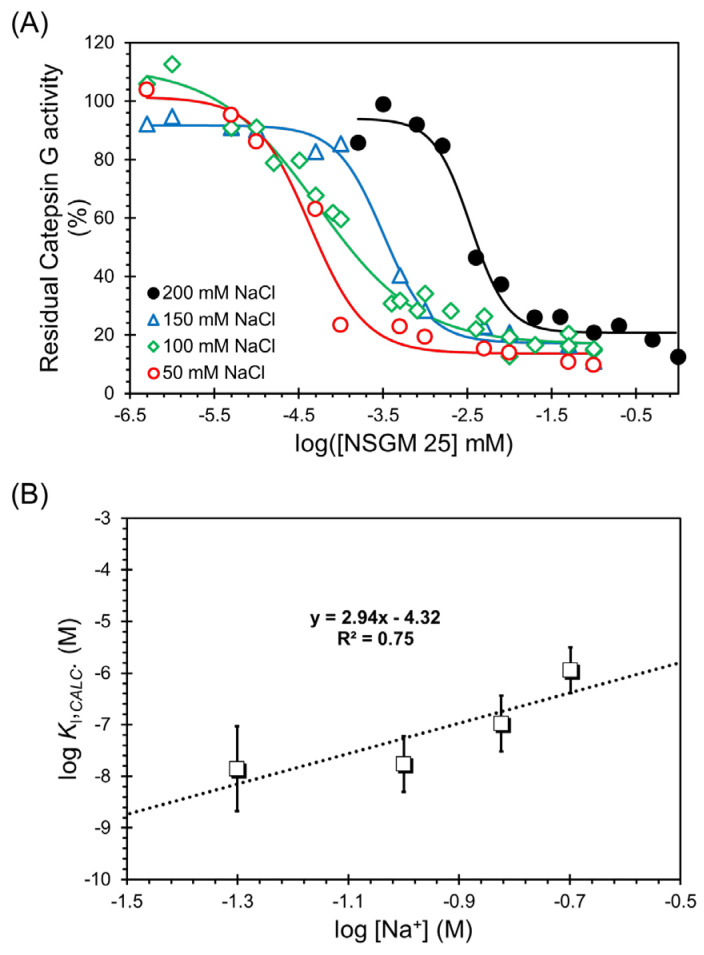
Salt dependence of CatG inhibition by NSGM **25**. (**A**) Salt-dependent direct inhibition of CatG by NSGM **25**. Data were obtained using substrate S-7388 in a chromogenic substrate hydrolysis assay. Solid lines represent sigmoidal dose–response analysis (Equation (1)) of the data to obtain IC_50_, ΔY%, and HS. Errors represent ± 1 S.E. (**B**) A double-log plot of the K_I_ calculated using Cheng–Rusoff equation for uncompetitive inhibition against the concentration of salt. Solid line shows linear analysis to obtain the intercept (log K_D,NONIONIC_) and slope (Z × ψ) (see Section 2.6), from which proportions of nonionic and ionic binding energies were derived.

To resolve these two types of contributing forces, we measured the IC_50_ of NSGM **25** at pH 7.4 and 37 °C as a function of the ionic strength of the buffer (Figure 6A). The dose dependence profiles clearly show a loss in potency, as expected. In fact, the IC_50_ values increased from 0.043 ± 0.01 µM to 3.42 ± 0.52 µM as NaCl concentration was increased from 50 mM to 200 mM (Table 4). This represents a substantial loss of ~80-fold in inhibition potency, and demonstrates that ionic forces are important to the CatG–NSGM **25** system.

The measured IC_50_s of our allosteric inhibitors are distinct from inhibition constants K_I_, which are thermodynamic constants. Cheng and Prusoff provided a mathematical foundation for the transformation of IC_50_s into K_I_s for uncompetitive inhibitors. In their formulation, K_I_ is equal to IC_50_ × [S]/(K_M_ + [S]) [43], which is directly applicable to our study. Thus, it becomes possible to utilize the linear double-log analysis described above for our NSGM **25**, which is an uncompetitive inhibitor. Figure 6B shows the double-log plot of K_I_ versus Na^+^ concentration, where the inhibition constants were calculated from the observed IC_50_ values. Linear regression yielded a slope of 2.94 and an intercept of −4.32 (Table 5). Whereas the former corresponds to an ionic binding energy of 4.88 kcal/mol at 37 °C and 100 mM salt, the latter yields a nonionic binding energy of 6.12 kcal/mol. These results show that the NSGM **25** interaction with CatG is driven by both electrostatic (~44%) and nonionic forces (~56%). Such important roles of two forces have not been observed earlier. More importantly, the higher nonionic component bodes well for the discovery of second-generation inhibitors with higher selectivity.

### 3.5. Impact of NSGM ***25*** on Human Plasma Clotting

**Figure 7:**
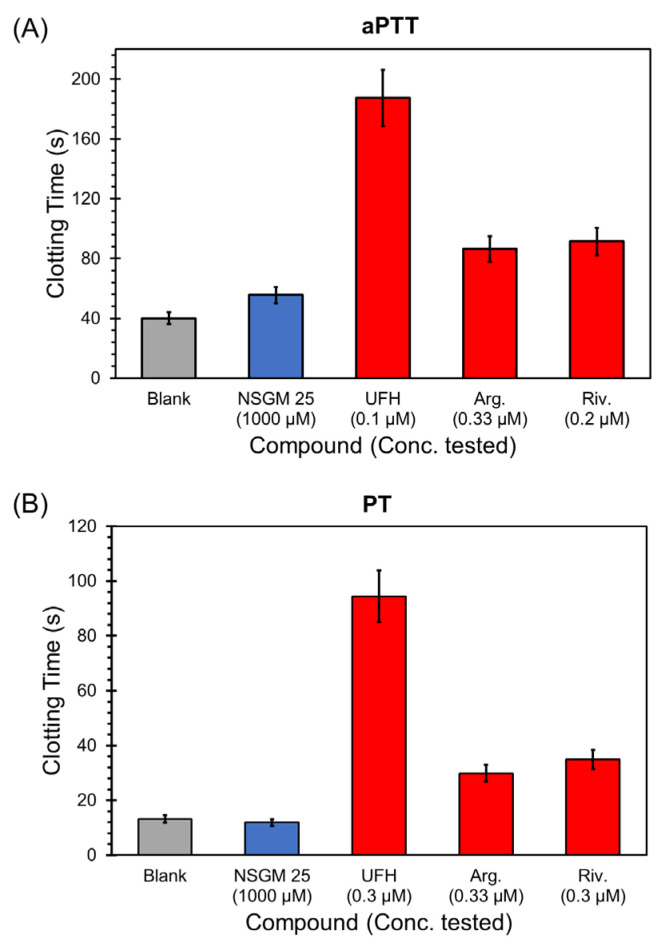
Impact of specific concentrations of NSGM 25 (blue bar) and the clinically-approved anticoagulants unfractionated heparin, argatroban, and rivaroxaban (red bars) on (**A**) activated partial thromboplastin time (aPTT) and (**B**) prothrombin time (PT). Grey bars are control experiments in which only buffer is used to measure clotting times.

Considering that NSGMs are functional mimetics of heparins, they have the potential to impact blood clotting by interfering with the activity of heparin-binding coagulation enzymes. For NSGM **25** to be useful as an anti-CatG agent in inflammatory conditions, it is important that it possesses a minimal risk of bleeding. In a previous study, we had reported that the inhibitory potencies of NSGM **25** against thrombin, factor Xa, factor XIa, factor IXa, and factor XIIa were poor (>150 µM) [47]. This implied a rather weak anticoagulant activity. However, a key test of clotting risk is the impact putative anticoagulants have on human plasma. To evaluate this, we measured the effect of NSGM **25** on activated partial thromboplastin time (aPTT) and prothrombin time (PT), two in vitro tests that are routinely used to assess anticoagulant potency [58]. Figure 7 shows the effect of specific concentrations of NSGM **25** and the clinically-approved anticoagulants unfractionated heparin (UFH), argatroban, and rivaroxaban on clotting time in the aPTT and PT assays. NSGM **25** did not impact either aPTT or PT at a concentration as high as 1 mM, which greatly exceeds its in vitro IC_50_ against CatG. In contrast, the clinically-approved anticoagulants doubled both the aPTT and PT at sub-micromolar concentrations (Table 6). Thus, NSGM **25** is not expected to present a high risk of bleeding, a significant advantage over current anticoagulants. Advanced studies will be needed to study the bleeding propensity of NSGM **25** in vivo.

## 4. Discussion

We pursued NSGM inhibition of CatG because of its importance in several inflammatory diseases, which has been challenging to fully understand because of the paucity of synthetic small molecule inhibitors, which may serve as high-quality chemical probes and eventually lead to therapeutics. In fact, it has been challenging to decipher the exact roles of different NSPs contributing to the protease–antiprotease balance, and high-quality chemical probes of CatG may aid in understanding the biology better. Nevertheless, in the context of therapy, it may be advantageous to also discover pan-protease inhibitor(s) that target CatG together with other pro-inflammatory proteases, e.g., HNE, PR3, and NSP4. One route to achieve this is to explore sulfated NSGMs because each of these proteases is known to bind to highly anionic biopolymers, e.g., GAGs and/or DNA [27,29,59,60,61].

In this study, we have shown that several structurally-defined, homogeneous NSGMs hold considerable promise as chemical biology probes of CatG, and may serve as early leads for therapeutics development. More specifically, the sulfated flavonoid class of NSGMs is especially promising because it presents two molecules with 53 nM (NSGM **25**) and 70 nM (NSGM **26**) inhibition potency. Discovery of such high-potency protease inhibitors in the first screen itself is a major achievement, when first attempts against other serine proteases, e.g., thrombin, factor Xa, factor XIa, etc., are considered [51,52]. This bodes well for further optimization of NSGM structure for potency as well as selectivity.

Although it may appear that sulfated benzofuran-based NSGMs are not worthy of further studies because of their moderate IC_50_s, these molecules may serve a very important function. Analysis of the drivers of their inhibition potency revealed that hydrophobicity and placement of hydrophobic groups were more important than the number of sulfate groups. In contrast, the sulfated flavonoid-based NSGMs emphasized the latter to be more important. Such divergent drivers of affinity could arise from different sites of binding on CatG. If so, it may be possible to use a fragment-based structural biology approach to develop a heterodimer from a sulfated benzofuran and sulfated flavonoid, thereby enhancing affinity as well as selectivity.

Michaelis–Menten studies revealed that NSGM **25** utilizes an uncompetitive inhibition mechanism. The simultaneous reduction in both K_M_ and V_MAX_ implies that **25** prefers the enzyme–substrate complex rather than the enzyme alone. This is an uncommon mechanism of inhibition because the majority of NSGMs studied to date have exhibited non-competitive inhibition mechanism [52]. Mechanistically, this phenomenon is extremely interesting because it implies that the binding of NSGM **25** in its allosteric site makes the enzyme recognize its substrate better, while at the same time not allowing it to perform its catalytic function. Structurally, it implies that the catalytic triad present in the active site is disrupted upon inhibitor complexation, but the other residues of the active site hold on to the substrate better.

The salt dependence studies provide another window into the recognition of NSGM **25** by CatG. These experiments show that NSGM **25** binding to CatG is driven by both electrostatic (~44%) and nonionic forces (~56%). The relatively high nonionic component of binding energy is interesting considering that electrostatics dominate the overall surface area of CatG. Because nonionic binding energy may arise from H-bonding, which is highly directional, and/or van der Waals forces, which have to be structurally complementary, the CatG–NSGM **25** interaction is expected to exhibit significant structural selectivity.

There are several advantages of pursuing NSGM **25** for further development as a probe and/or therapeutic agent. First, in contrast to GAGs and heparin, it is a small, homogeneous compound that can be obtained fairly readily via synthetic means using commercially-available raw materials [47]. NSGM **25** also works via an allosteric process and, because allosteric sites on proteins are less conserved than orthosteric sites, there is a reduced possibility of off-target effects. This is also evident in its inability to inhibit coagulation proteases and impact on plasma clotting. More importantly, previous studies with NSGM **25** show its excellent inhibition of HNE (IC_50_ ~230 nM) [62] and moderate inhibition of human plasmin (IC_50_ ~6.3 µM) [47], both of which are known to be pro-inflammatory [63,64,65]. While HNE potentiates inflammation via mechanisms similar to CatG [44], plasmin enhances multiple pro-inflammatory responses, including the generation of pro-inflammatory fibrin degradation products, activation of the complement, and activation of matrix metalloproteinases [66]. Thus, NSGM **25′**s multiple inflammatory proteases activity, in the absence of impact on coagulation proteases, makes it a very promising inhibitor for further development. As with any such early study, this promise should always be balanced by cross-checking reactivities with other related proteases, such as those of the complement cascade, which may or may not interfere with the functions of such novel probes.

## Figures and Tables

**Table 1:** Direct Inhibition of Human CatG by Sulfated Benzofuran Molecules.

NSGM	IC_50_ (µM) ^a^	ΔY (%) ^b^	HS ^c^
**1**	>50	ND	ND
**2**	>50	ND	ND
**3**	>50	ND	ND
**4**	48.3 ± 6.8 ^d^	96 ± 6	1.3 ± 0.2
**5**	5.5 ± 0.8	93 ± 5	5.3 ± 1.9
**6**	~50	ND	ND
**7**	>50	ND	ND
**8**	27.0 ± 3.1	101 ± 5	1.7 ± 0.3
**9**	45.5 ± 3.3	99 ± 4	2.5 ± 0.4
**10**	29.0 ± 6.5	97 ± 10	1.5 ± 0.5
**11**	>50	ND	ND
**12**	19 ± 12	87 ± 4	8.6 ± 5.1
**13**	42.1 ± 4.2	90 ± 5	2.3 ± 0.5
**14**	17.3 ± 2.8	96 ± 7	1.6 ± 0.4
**15**	>50	ND	ND
**16**	126 ± 11	115 ± 4	1.9 ± 0.3
**17**	65 ± 13	76 ± 7	3.1 ± 1.3

^a^ IC_50_ values, ^b^ ΔY, and HS; ^c^ HSs were obtained by non-linear regression of direct CatG inhibition by the NSGMs. ^d^ Error represents ± 1 S.E. Please refer to the Section 2.4 to review the definitions of ΔY and HS.

**Table 2:** Direct Inhibition of Human CatG by Sulfated Flavonoid Dimers.

NSGM	IC_50_ (µM) ^a^	Δ Y (%) ^b^	HS ^c^
**18**	4.7 ± 0.7 ^d^	81 ± 7	1.5 ± 0.3
**19**	3.3 ± 0.3	82 ± 4	3.1 ± 1.0
**20**	1.5 ± 0.2	89 ± 4	1.4 ± 0.2
**21**	10.3 ± 4.8	81 ± 7	1.3 ± 0.8
**22**	1.3 ± 0.2	105 ± 7	1.2 ± 0.2
**23**	3.4 ± 0.2	103 ± 4	4.1 ± 1.0
**24**	0.21 ± 0.04	94 ± 9	1.5 ± 0.5
**25**	0.05 ± 0.01	95 ± 7	0.7 ± 0.1
**26**	0.07 ± 0.01	101 ± 5	1.2 ± 0.2
**27**	0.15 ± 0.06	77 ± 10	0.9 ± 0.4
**28**	0.18 ± 0.04	77 ± 5	0.9 ± 0.2
**29**	0.14 ± 05	110 ± 16	1.1 ± 0.1
**30**	18.9 ± 2.2	87 ± 5	2.1 ± 0.5

^a^ IC_50_ values, ^b^ ΔY, and HS; ^c^ HSs were obtained by non-linear regression of direct CatG inhibition by the NSGMs. ^d^ Error represents ± 1 S.E.

**Table 3:** Michaelis–Menten Kinetics of CatG Hydrolysis of Chromogenic Substrate (S-7388) in the Presence of NSGM **25** ^a^.

[NSGM 25] (nM)	K_M_ (mM)	V_MAX_ (mAu/min)
**0**	2.26 ± 0.28 ^b^	70.0 ± 5.5
**50**	2.18 ± 0.27	52.9 ± 4.1
**100**	1.31 ± 0.06	31.0 ± 0.8
**150**	0.87 ± 0.2	21.1 ± 2.1
**200**	0.80 ± 0.11	17.0 ± 1.0
**400**	0.77 ± 0.30	12.4 ± 2.1

^a^ K_M_ and V_MAX_ values were measured as described in the experimental section. mAU indicates milliabsorbance units. ^b^ Error represents ± 1 S.E.

**Table 4:** Salt-Dependence of CatG Inhibition by NSGM **25**.

[NaCl]	IC_50_ (nM) ^a^	ΔY (%) ^b^	HS ^c^	K_I_ (nM) ^d^
**50**	42.7 ± 10	87.7 ± 7.3 ^b^	1.3 ± 0.4	10.9 ± 3.9
**100**	52.6 ± 12.2	94.9 ± 17.9	0.7 ± 0.1	19.2 ± 5.3
**150**	318.6 ± 44.5	74.4 ± 2.9	1.6 ± 0.3	148 ± 54
**200**	3453 ± 517	73.2 ± 4.9	2.0 ± 0.5	1671 ± 480

^a^ IC_50_ values, ^b^ ΔY, and HS; ^c^ HS were obtained by non-linear regression of direct CatG inhibition by NSGM **25**. ^d^ Calculated using Cheng–Prusoff equation [54].

**Table 5:** Calculated energies of binding of CatG and NSGM **25**.

Slope	Intercept	K_I,NON-IONIC_ (µM)	ΔG_NON-IONIC_ (kcal/mol)	ΔG_NON-IONIC_ (%)	ΔG_IONIC_ (%)
2.94	−4.32	158.5	6.12	55.6	44.4

Slope and intercept were calculated from linear regression analysis of log K_I_ versus log[Na^+^] using the equation log K_I_ = log K_I,NONIONIC_ + Zψ log [Na^+^] [53].

**Table 6:** Impact of NSGM **25** on aPTT and PT ^a^.

Sample	aPTT (EC_2X_) (µM)	PT (EC_2X_) (µM)
NSGM **25**	>1000	>1000
UFH	0.045	0.170
Argatroban	0.34	0.36
Rivaroxaban	0.12	0.15

^a^ Concentrations of molecules required to double the aPTT or the PT of pooled human plasma.

## Data Availability

Not applicable.
